# Cooling Effectiveness of a Data Center Room under Overhead Airflow via Entropy Generation Assessment in Transient Scenarios

**DOI:** 10.3390/e21010098

**Published:** 2019-01-21

**Authors:** Luis Silva-Llanca, Marcelo del Valle, Alfonso Ortega, Andrés J. Díaz

**Affiliations:** 1 Instituto de Investigación Multidisciplinario en Ciencia y Tecnología, Departamento de Ingeniería Mecánica, Universidad de La Serena, Benavente 980, La Serena 1720170, Chile; 2 Laboratory for Advanced Thermal and Fluid Systems, Mechanical Engineering Department, Villanova University, 800 East Lancaster Avenue, Villanova, PA 19085, USA; 3 Facultad de Ingeniería y Ciencias, Escuela de Ingeniería Industrial, Universidad Diego Portales, Av. Ejército 441, Santiago 8370191, Chile

**Keywords:** data center heating, ventilation, and air conditioning (HVAC), overhead cooling, entropy generation, dynamic modeling

## Abstract

Forecasting data center cooling demand remains a primary thermal management challenge in an increasingly larger global energy-consuming industry. This paper proposes a dynamic modeling approach to evaluate two different strategies for delivering cold air into a data center room. The common cooling method provides air through perforated floor tiles by means of a centralized distribution system, hindering flow management at the aisle level. We propose an idealized system such that five overhead heat exchangers are located above the aisle and handle the entire server cooling demand. In one case, the overhead heat exchangers force the airflow downwards into the aisle (Overhead Downward Flow (ODF)); in the other case, the flow is forced to move upwards (Overhead Upward Flow (OUF)). A complete fluid dynamic, heat transfer, and thermodynamic analysis is proposed to model the system’s thermal performance under both steady state and transient conditions. Inside the servers and heat exchangers, the flow and heat transfer processes are modeled using a set of differential equations solved in MATLAB™ 2017a. This solution is coupled with ANSYS-Fluent™ 18, which computes the three-dimensional velocity, temperature, and turbulence on the Airside. The two approaches proposed (ODF and OUF) are evaluated and compared by estimating their cooling effectiveness and the local Entropy Generation. The latter allows identifying the zones within the room responsible for increasing the inefficiencies (irreversibilities) of the system. Both approaches demonstrated similar performance, with a small advantage shown by OUF. The results of this investigation demonstrated a promising approach of data center on-demand cooling scenarios.

## 1. Introduction

Data center thermal management is generally accomplished by providing cold air from a raised floor into the server aisle. In some designs, the cooling is aided by the strategic placing of heat exchanger arrangements: overhead, in-row, rear-door. Limited academic work exists on each system, where most of the available information is provided by the equipment suppliers in catalogs and manuals.

The majority of the authors center their efforts on cases where centralized CRAH units provide the cold air. The air is then ducted and distributed to the data center room aisles [1,2,3,4]. This approach, which should be considered a centralized cooling application, suffers the same control issues as underfloor centralized cooling. If a hot spot appears in the data center room, operators must increase the amount of air delivered by the CRAH units, overprovisioning the complete room, and reducing cooling system energy efficiency.

Hong-Koo et al. [1] used the temperature cooling performance, the maximum temperature cooling performance, and the cooling performance ratio to evaluate the different air cooling configurations. Nakao et al. [2] used a “differential temperature ratio” as a way to evaluate the efficiency of the different systems using experimental data. The differential temperature ratio is a previous version of what is currently known as the Supply Heat Index (SHI), which evaluates recirculation inside the data center room. Sorell et al. [3] compared underfloor and overhead air delivery systems by calculating the SHI and the Return Heat Index (RHI) metrics.

A scarce number of publications have used overhead cooling as a distributed cooling system. Some authors used the overhead modules as a secondary cooling source to prevent hot spots. Heydari and Sabounchi [5] and Heydari [6] used steady state CFD simulations to model overhead cooling systems in combination with CRAH units. The studies showed that overhead cooling systems are effective in the presence of hot spots when used as an additional cold air supplier, especially in high power density cases.

Wu [7] used CFD simulations to compare overhead systems’ performance with other commonly used data center cooling technologies. The author concluded that overhead systems can prevent recirculation at the top of the cabinets in open configurations. For the cabinets near the top of the rack, inadequate provisioning of air was observed due to the high momentum in the cold air jet leaving the overhead system. The CFD data indicated that overhead systems can handle high power rack densities, although they presented difficulties in building enough static pressure in low power densities. Herrlin and Belady [8] compared three different configurations using CFD simulations: a bottom-up (raised floor configuration), top-down (modular overhead equipment placed on top of racks), and reverse-tile system (overhead equipment on top of the cold aisle). The authors used the Rack Cooling Index metric (RCI) to evaluate the best possible configuration. The results showed that the modular top-down configuration gives the best results with RCI near 100% with almost total absence of recirculation, thanks to “gravity-assisted” mixing in the cold aisle.

This work focuses on the modeling and comparison of two different overhead cooling configurations: Overhead Downward Flow (ODF) and Overhead Upward Flow (OUF). In both cases, the overhead systems are used as the sole source of cooling for the data center room. Energy efficiency is examined using the Entropy Generation Rate to locate and quantify the inefficiencies in the cooling system. Shah et. al [9] was the first author to introduce this technique to data center applications.

Kock and Herwig [10,11] developed a formulation to study turbulent flows from a second law perspective and demonstrated that turbulent fluctuations significantly enhance irreversibilities. Herwig [12] presented an overview of the state-of-the-art in turbulent flows, which we believe to be highly applicable to data center air flows. Computational fluid dynamics has been combined with the second law of thermodynamics to determine inefficient practices [9,13,14,15,16,17]. Silva-Llanca et al. [14] compared two numerical models for the calculation of Exergy Destruction in perimeter-cooled data center turbulent flows using data obtained from CFD simulation. They found that the Entropy Generation due to viscous dissipation (usually neglected in most applications) was approximately 36% of the total in the airspace. The Entropy Generation due to heat conduction emerged due to the pre-mixing of cold and hot air streams in the cold aisle.

## 2. Motivation and Goals

The application of overhead systems in data centers raises interesting questions that, to the best of our knowledge, remain unaddressed in the literature. One example is the lack of conclusiveness as to what is the more energy-efficient strategy to deploy overhead cooling, either injecting downward cold air into the aisle or removing hot airflow upwardly from the aisle. Distributed cooling such as overhead heat exchangers can advantageously operate when combined with dynamic on-demand cooling applications, in such a way that cooling can be delivered to the right location and at the right moment.

In this work, we focus on quantifying the energy efficiency of downward and upward overhead airflow, as well as the transient behavior of such systems. Our analysis incorporates the simultaneous unsteady response in the heat exchangers, servers, and the cooling flow. We will demonstrate the importance of each component in the cooling dynamics and how relevant it is to contain the aisle.

This paper showcases the early efforts of an ambitious project that intends to advance the research on the thermal energy efficiency of dynamically-operated data center HVAC systems. At this first stage, we aim to develop transient numerical models of the contributing components inside the server room. With this in mind, we created an idealized data center geometry to apply first and second law thermodynamic principles, so that we can systematically examine the system’s thermal efficiency.

## 3. Mathematical Model and Numerical Implementation

**Figure 1:**
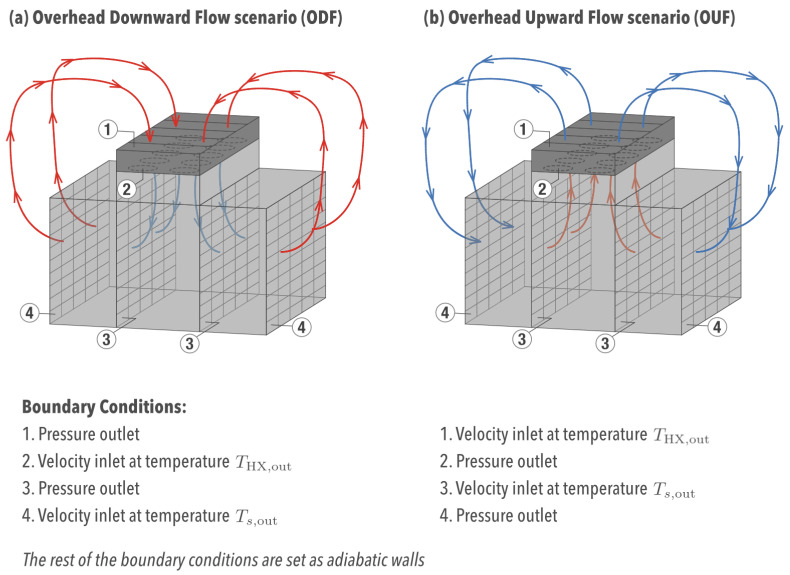
Schematic of the room and boundary conditions for the two approaches: (**a**) downward flow and (**b**) upward flow.

Figure 1 depicts the two overhead cooling scenarios to be studied in this work: (a) Overhead Downward Flow (ODF), or the cold aisle approach, and (b) Overhead Upward Flow (OUF), or the hot aisle approach.

**Figure 2:**
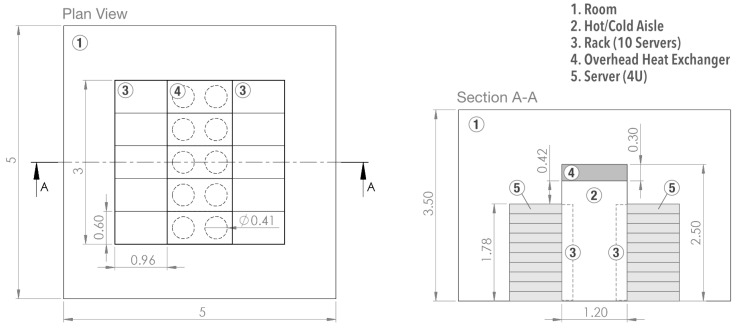
Two-dimensional drawing for the data center room (dimensions in meters).

The data center room is comprised of 10 racks with 10 servers (4U) each and five overhead heat exchangers placed at the aisle ceiling. Figure 2 shows a scaled engineering drawing of the physical domain. The problem is studied dynamically (transient) and undergoes the following assumptions:

- The room transfers no heat to the outside (adiabatic walls),
- Fully-contained aisle, in other words, air only flows through servers and heat exchangers,
- Heat conduction allowed through the aisle containment walls,
- Fluid flow in the turbulent regime,
- Constant server heat dissipation of 1 kW, for a total of 100 kW of required overhead cooling.

### 3.1. Airside

The Airside is comprised of the room and hot/cold aisle sections (Figure 2) and it is assumed to be in the turbulent regime, approximated by the *k*-ε model as follows [18]:

Continuity:

(1) $$\begin{array}{ccc} \frac{\partial {\bar{u}}_{i}}{\partial x_{i}} & = & 0 \end{array}$$

Momentum:

(2) $$\begin{array}{ccc} \frac{\partial {\bar{u}}_{i}}{\partial t}+\frac{\partial {\bar{u}}_{i}{\bar{u}}_{j}}{\partial x_{j}} & = & -\frac{1}{\rho }\frac{\partial \bar{p}}{\partial x_{i}}+\frac{\partial }{\partial x_{i}}\left[\nu \left(\frac{\partial {\bar{u}}_{i}}{\partial x_{j}}+\frac{\partial {\bar{u}}_{j}}{\partial x_{i}}\right)-\bar{u_{i}^{\prime }u_{j}^{\prime }}\right] \end{array}$$

Energy:

(3) $$\begin{array}{ccc} \frac{\partial \bar{T}}{\partial t}+{\bar{u}}_{j}\frac{\partial \bar{T}}{\partial x_{j}} & = & \frac{\partial }{\partial x_{j}}\left(\alpha \frac{\partial \bar{T}}{\partial x_{j}}-\bar{u_{j}^{\prime }T^{\prime }}\right) \end{array}$$

Turbulent kinetic energy:

(4) $$\begin{array}{ccc} \frac{\partial k}{\partial t}+{\bar{u}}_{j}\frac{\partial k}{\partial x_{j}} & = & \frac{\partial }{\partial x_{j}}\left[\left(\nu +\frac{\nu_{T}}{\sigma_{k}}\right)\frac{\partial k}{\partial x_{j}}\right]+2\nu_{t}S_{ij}:S_{ij}-\varepsilon \end{array}$$

Rate of viscous dissipation:

(5) $$\begin{array}{ccc} \frac{\partial \varepsilon }{\partial t}+{\bar{u}}_{j}\frac{\partial \varepsilon }{\partial x_{j}} & = & \frac{\partial }{\partial x_{j}}\left[\left(\nu +\frac{\nu_{T}}{\sigma_{\varepsilon }}\right)\frac{\partial \varepsilon }{\partial x_{j}}\right]+C_{1\varepsilon }\frac{\varepsilon }{k}2\nu_{t}\left(S_{ij}:S_{ij}\right)-C_{2\varepsilon }\frac{\varepsilon^{2}}{k} \end{array}$$

where $\nu_{t}=C_{\mu }k^{2}/\varepsilon$ is the turbulent eddy viscosity and *S* is the rate-of-strain tensor.

### 3.2. Server Transient Model

We approximate the transient response in the servers via the lumped approach introduced by Erden et al. [19] and Demetriou et al. [20]. They assumed the server as a black box with a constant cooling flow rate and no internal heat generation:

(6) $$\begin{array}{ccc} \theta (t) & = & \theta \left(0\right)e^{-t/\tau } \end{array}$$

(7) $$\begin{array}{ccc} \theta & = & \frac{{\dot{Q}}_{s}}{K}-T_{s}+T_{s,\mathrm{in}} \end{array}$$

The heat transfer in the server is modeled by approximating its behavior to that of a heat exchanger, where an average temperature $T_{s}$ is assigned such that we can introduce the heat exchanger effectiveness as a function of the Number of Transfer Units (NTU). The thermal resistance associated with the server, *K*, is determined as a function of the server effectiveness, $\xi_{s}$, and the flow capacity, ${\dot{C}}_{a}={\dot{m}}_{a}c_{p,a}$:

(8) $$\begin{array}{ccc} K & = & \xi_{s}{\dot{C}}_{a} \end{array}$$

(9) $$\begin{array}{ccc} \xi_{s} & = & 1-e^{-\mathrm{NTU}}=f\left(\mathrm{CFM}\right) \end{array}$$

(10) $$\begin{array}{ccc} T_{s,\mathrm{in}}-T_{s,\mathrm{out}} & = & \xi_{s}\left(T_{s,\mathrm{in}}-T_{s}\right) \end{array}$$

Experimental data presented in [20] were used to extract the time constant τ and the server effectiveness $\xi_{s}$ for different volumetric flow rates in a 4U rack server.

### 3.3. Heat Exchanger Transient Model

The overhead system has five air-to-liquid cross-flow heat exchangers. The dynamic behavior is dependent on the thermal inertia of metals and fluids, as well as the geometric arrangement. The unsteady response of cross-flow heat exchangers has been extensively investigated starting in the 1950s. Because this is a multi-dimensional and unsteady physical situation, no analytical and compact solution exits.

Del Valle and Ortega [21] investigated—numerically and experimentally—the dynamic response of a cross-flow heat exchanger. The experiment was designed so that different temperature perturbations can be introduced into the system, with their subsequent transient outputs. The numerical aspect was carried out using a finite difference algorithm, demonstrating excellent agreement with the experimental data. This validated model was then used to feed data into an Artificial Neural Network (ANN) compact model. This way, the authors generated rapid and accurate predictions of the transient phenomena, greatly reducing computational costs by eliminating the need for CFD.

The air stream, water stream, and wall were modeled via individual energy balances, forming a system of three unsteady non-linear differential equations. The model was subject to the following assumptions: (i) negligible heat losses, (ii) no thermal energy sources or sinks, (iii) single-phase flow, (iv) uniform heat transfer coefficients, and (v) uniformly-distributed wall thermal resistance in the entire heat exchanger with negligible axial heat conduction [22].

The non-dimensionalized set of equations yields [23]:

(11) $$\begin{array}{ccc} \frac{\partial T_{\mathrm{wall}}^{*}}{\partial t^{*}}+\left(1+R\right)T_{\mathrm{wall}}^{*} & = & T_{a}^{*}+RT_{w}^{*} \end{array}$$

(12) $$\begin{array}{ccc} V_{a}\frac{\partial T_{a}^{*}}{\partial t^{*}}+\frac{\partial T_{a}^{*}}{\partial x^{*}}+\left(T_{a}^{*}-T_{\mathrm{wall}}^{*}\right) & = & 0 \end{array}$$

(13) $$\begin{array}{ccc} V_{w}\frac{\partial T_{w}^{*}}{\partial t^{*}}+\frac{\partial T_{w}^{*}}{\partial x^{*}}+\left(T_{w}^{*}-T_{\mathrm{wall}}^{*}\right) & = & 0 \end{array}$$

where:

(14) $$\begin{array}{c} x^{*}=\frac{{\left(hA\right)}_{a}\;x}{{\left(\dot{m}c\right)}_{a}\;L_{a}}\;y^{*}=\frac{{\left(hA\right)}_{w}\;y}{{\left(\dot{m}c\right)}_{w}\;L_{w}}\;T^{*}=\frac{T-T_{w,\mathrm{in}}}{T_{a,\mathrm{in}}-T_{w,\mathrm{in}}} \end{array}$$

(15) $$\begin{array}{c} t^{*}=\frac{{\left(hA\right)}_{a}}{{\left(mc\right)}_{\mathrm{wall}}}\;V_{w}=\frac{{\left(\dot{m}c\right)}_{w}}{{\left(mc\right)}_{\mathrm{wall}}}\frac{L_{w}}{u_{w}}\;V_{a}=\frac{{\left(\dot{m}c\right)}_{a}}{{\left(mc\right)}_{\mathrm{wall}}}\frac{L_{a}}{u_{a}} \end{array}$$

(16) $$\begin{array}{c} R=\frac{{\left(hA\right)}_{w}}{{\left(hA\right)}_{a}}\;\mathrm{NTU}={\left\{{\left(\dot{m}c\right)}_{\min}\left[\frac{1}{{\left(hA\right)}_{a}}+\frac{1}{{\left(hA\right)}_{w}}\right]\right\}}^{-1} \end{array}$$

The subindices *a* and *w* correspond to air and water, respectively. The complete mathematical formulation may be found in [21].

### 3.4. Entropy Generation

**Figure 3:**
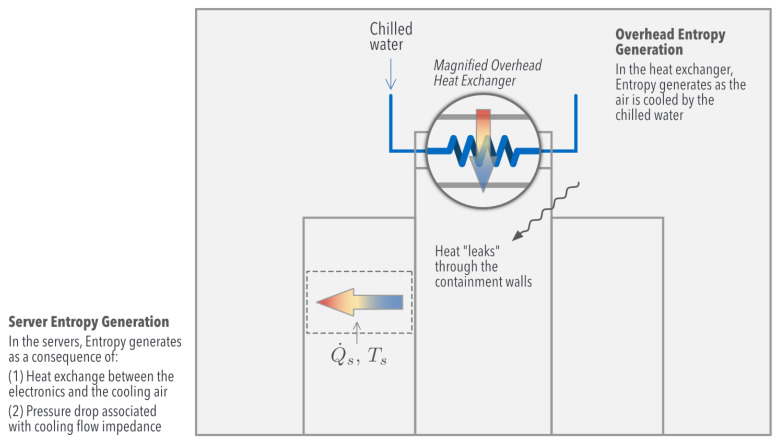
Schematic representation of the phenomena that destroys entropy during the cooling process.

One consequence of the second law of thermodynamics is the concept of Entropy Generation, which quantifies loses (irreversibilities) that take place in a process, in this case being data center cooling (Figure 3). The processes considered in this work generate entropy mainly from three sources:

1. Servers: heat transfer between the electronics and the cooling air; pressure drop as the air flows through the server, undergoing significant impedance
2. Overhead heat exchangers: heat transfer between the chilled water and the airflow
3. Airside: the temperature difference between the room and the aisle induces heat transfer through the containment walls; turbulent viscous dissipation of momentum becomes non-negligible

The transient entropy balance in the servers and overhead system yields:

(17) $$\begin{array}{ccc} {\dot{S}}_{\mathrm{gen},s} & = & \frac{dS}{dt}+\dot{m}\left[c_{p}\ln\left(\frac{T_{s,\mathrm{out}}}{T_{s,\mathrm{in}}}\right)-R\ln\left(\frac{P_{s,\mathrm{out}}}{P_{s,\mathrm{in}}}\right)\right]-\frac{{\dot{Q}}_{s}}{T_{s}} \end{array}$$

(18) $$\begin{array}{ccc} {\dot{S}}_{\mathrm{gen},\mathrm{HX}} & = & \frac{dS}{dt}+\dot{m}c_{p}\ln\left(\frac{T_{\mathrm{HX},\mathrm{out}}}{T_{\mathrm{HX},\mathrm{in}}}\right)+\frac{{\dot{Q}}_{\mathrm{HX}}}{T_{w}} \end{array}$$

The turbulent fluid flow and temperature distribution in the Airside require expressing the Entropy Generation at the infinitesimal level. A procedure first introduced by Kock and Herwig [10] was successfully used by Silva-Llanca et al. [14] in the simulation of a legacy data center, where the Entropy Generation rate per unit volume was calculated as:

(19) $$\begin{array}{cc} {\dot{S}}_{\mathrm{gen},\mathrm{airside}}^{\prime \prime \prime } & =\underset{{\dot{S}}_{\mathrm{gen},\bar{D}}^{\prime \prime \prime }}{\underset{︸}{\frac{\mu }{\bar{T}}\left\{2\left[{\left(\frac{\partial {\bar{u}}_{x}}{\partial x}\right)}^{2}+{\left(\frac{\partial {\bar{u}}_{y}}{\partial y}\right)}^{2}+{\left(\frac{\partial {\bar{u}}_{z}}{\partial z}\right)}^{2}\right]+{\left(\frac{\partial {\bar{u}}_{x}}{\partial y}+\frac{\partial {\bar{u}}_{y}}{\partial x}\right)}^{2}+{\left(\frac{\partial {\bar{u}}_{x}}{\partial z}+\frac{\partial {\bar{u}}_{z}}{\partial x}\right)}^{2}+{\left(\frac{\partial {\bar{u}}_{y}}{\partial z}+\frac{\partial {\bar{u}}_{z}}{\partial y}\right)}^{2}\right\}}}\\ & +\underset{{\dot{S}}_{\mathrm{gen},D^{\prime }}^{\prime \prime \prime }}{\underset{︸}{\frac{\rho \varepsilon }{\bar{T}}}}+\underset{{\dot{S}}_{\mathrm{gen},\bar{C}}^{\prime \prime \prime }}{\underset{︸}{\frac{\lambda }{{\bar{T}}^{2}}\left[{\left(\frac{\partial \bar{T}}{\partial x}\right)}^{2}+{\left(\frac{\partial \bar{T}}{\partial y}\right)}^{2}+{\left(\frac{\partial \bar{T}}{\partial z}\right)}^{2}\right]}}+\underset{{\dot{S}}_{\mathrm{gen},C^{\prime }}^{\prime \prime \prime }}{\underset{︸}{\frac{\alpha_{t}}{\alpha }\frac{\lambda }{{\bar{T}}^{2}}\left[{\left(\frac{\partial \bar{T}}{\partial x}\right)}^{2}+{\left(\frac{\partial \bar{T}}{\partial y}\right)}^{2}+{\left(\frac{\partial \bar{T}}{\partial z}\right)}^{2}\right]}} \end{array}$$

where $\alpha_{t}=\nu_{t}/{\mathrm{Pr}}_{t}$ is the turbulent thermal diffusivity, λ is thermal conductivity, and ${\mathrm{Pr}}_{t}$ is the turbulent Prandtl number.

The four terms in (19) are individualized as:

1. ${\dot{S}}_{\mathrm{gen},\bar{D}}^{\prime \prime \prime }$: Entropy Generation by direct dissipation.
2. ${\dot{S}}_{\mathrm{gen},D^{\prime }}^{\prime \prime \prime }$: Entropy Generation by turbulent dissipation.
3. ${\dot{S}}_{\mathrm{gen},\bar{C}}^{\prime \prime \prime }$: Entropy Generation by heat conduction.
4. ${\dot{S}}_{\mathrm{gen},C^{\prime }}^{\prime \prime \prime }$: Entropy Generation by heat transfer with fluctuating temperature gradients.

The total Entropy Generation rate in the Airside is obtained by integrating Equation (19):

(20) $$\begin{array}{c} {\dot{S}}_{\mathrm{gen},\mathrm{airside}}=\underset{\mathrm{airside}}{\;\int \int \int \;}{\dot{S}}_{\mathrm{gen},\mathrm{airside}}^{\prime \prime \prime }\;dV \end{array}$$

### 3.5. Numerical Procedure

A complete three-dimensional simulation of the fluid dynamics and heat transfer process that takes place within the Airside (aisle + room) was conducted using a transient turbulent model (*k*-ε) in ANSYS-Fluent^TM^. The velocity and pressure fields were coupled using the SIMPLE algorithm, and the nodes were interpolated via a second order upwind scheme.

The heat transfer within the servers and heat exchangers was modeled as described by Equations (6)–(18) via a finite difference scheme solved in MATLAB^TM^. We considered air as an ideal gas and assumed both the impedance and fan curves as a quadratic function of the mass flow rate, obtaining server volumetric flow rates of approximately 50 CFM.

After one time step computed in Fluent, MATLAB uses the obtained results as inputs to solve the already mentioned equations and to update the boundary conditions in Fluent—depicted in Figure 1—for the next time step. For example, any given server exit temperature $T_{s,\mathrm{out}}$ corresponds to an inlet temperature at which the airflow enters the CFD numerical domain (Figure 1). The same idea applies to the overhead flow.

Since the servers and overhead heat exchangers are modeled outside Fluent, the Airside numerical domain considers those zones as void volumes.

The solution of the complex system of algebraic and differential equations was carried out algorithmically as follows:

1. Fluent computes the Airside turbulent flow and moves the solution forward by one time step
2. The airflow data are used as inputs to the servers and heat exchangers
3. MATLAB solves the servers and heat exchangers
4. MATLAB output data become inlet boundary conditions for Fluent, and the fluid flow is updated
5. Go back to Step 1 under these new conditions

To enhance the early numerical convergence (i.e., t<5 s), an s-shaped function that ranged from 0–1 was utilized to weight the air mass flow rate.

The temporal discretization considers a constant time step δt=0.005 s employed until the flow developed t≤7 s, for upward and downward cooling. When t>7 s and until the steady state was reached (t≈3400 s), δt was varied dynamically from 0.1–1 s. This way, we accelerated the simulation and saved CPU time.

### 3.6. Grid Independence

**Figure 4:**
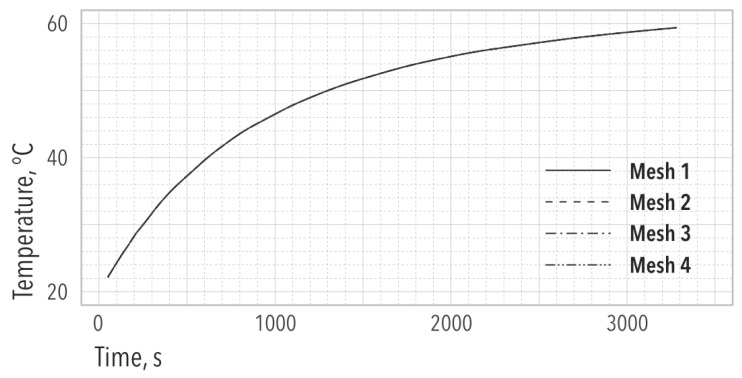
Server average temperature evolution for different grid sizes.

A mesh validation was obtained by comparing the server and the Airside Entropy Generation in four different meshes (Figure 4 and Table 1). Within the servers, Figure 4 shows that each of the four selected meshes can predict the temperature response with equal accuracy. This is also observed in Table 1 for the total Entropy Generation. Nonetheless, when considering the Airside, Table 1 shows that only Mesh 3 is able to capture the Entropy Generation with less than 3% error compared to the finest mesh. Therefore, Mesh 3 was chosen to carry out the numerical analysis.

## 4. Steady State Analysis

The following analysis intends to elucidate the contribution of both fluid mechanics and heat transfer to the increase in the thermodynamic irreversibilities associated with the use of overhead cooling systems.

**Figure 5:**
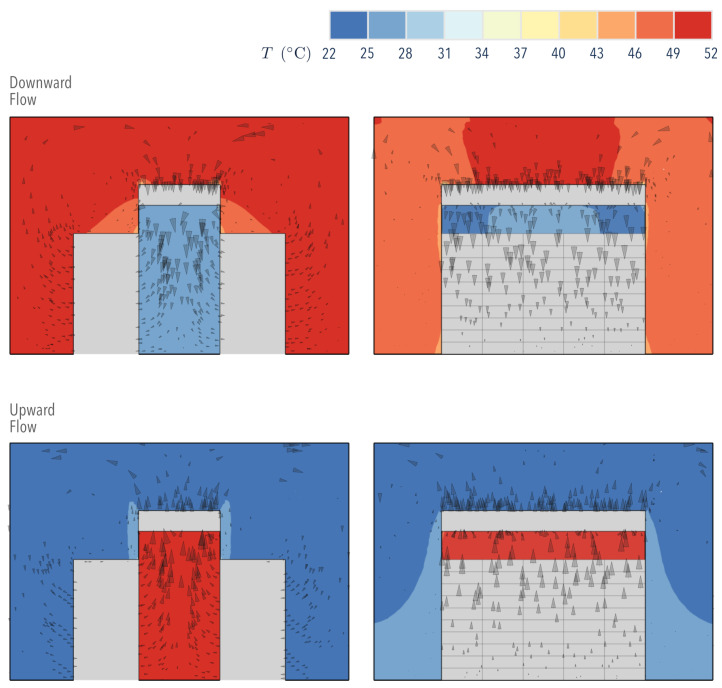
Airside temperature and velocity distributions; front and side view, middle plane.

**Figure 6:**
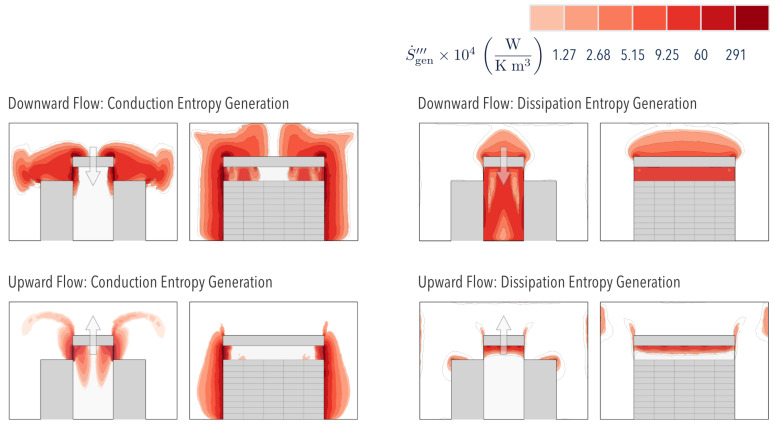
Conduction and Dissipation Entropy Generation distribution inside the room for the two approaches. Arrow indicates flow direction in the overhead heat exchanger.

Figure 5 shows the temperature distribution within the room with either ODF or OUF cooling. The results show a uniform distribution in which temperature gradients are located near the walls that separate the aisle from its surroundings (containment). These gradients are responsible for creating zones of maximum local thermal Entropy Generation (${\dot{S}}_{\mathrm{gen},\mathrm{airside}}^{\prime \prime \prime }$), as observed in Figure 6 for both ODF and OUF cooling modes.

Thermal irreversibilities in the room were greater than in the aisle when ODF cooling was used. This can be attributed to the higher amount of thermal energy contained in the surrounding air, an inefficient form of energy. Within the aisle, the local thermal Entropy Generation was higher at the wall in direct contact with the surroundings, in which the hot air conducted heat to the cold air. Away from this interface, both cooling modes showed that the temperature became more uniform; therefore, the thermal Entropy Generation considerably decreased.

Figure 6 shows two plumes in the OUF cooling mode, suggesting the presence of a cold fluid vortex created when the cold air flows upwardly after leaving the heat exchanger. Here, the cold fluid absorbed the heat from the stagnant hot air heated at the dividing wall.

Figure 6 also depicts the local Entropy Generation due to fluid friction ${\dot{S}}_{\mathrm{gen},D}$. The results indicate that higher inefficiencies existed in diverging flows (ODF), which is attributed to a larger pressure drop (as demonstrated in [14]).

### Entropy Generation in an Idealized Case

We now compare all the sources of thermal irreversibility inside the room, for which the thermodynamic cycle of Figure 3 is considered. We intend to identify the parameters that control the thermal Entropy Generation of the system, as well as the sources responsible for its increase. Here, the second law establishes that:

(21) $$\begin{array}{ccc} {\dot{S}}_{\mathrm{gen}} & = & \dot{m}c_{p}\ln\left(\frac{T_{s,\mathrm{out}}}{T_{s,\mathrm{in}}}\right)+\dot{m}c_{p}\ln\left(\frac{T_{\mathrm{HX},\mathrm{out}}}{T_{\mathrm{HX},\mathrm{in}}}\right)-\frac{{\dot{Q}}_{s}}{T_{s}}+\frac{{\dot{Q}}_{\mathrm{HX}}}{T_{w}} \end{array}$$

The first two terms on the right-hand side of Equation (21) consider the Entropy Generation associated with the heat transfer between the aisle and room, whereas the last two terms consider the heat transfer that takes place in the servers and overhead unit, respectively.

Table 2 shows the total Entropy Generation within the servers, overhead, and Airside. The effect of containment greatly reduced the Entropy Generation in the Airside when compared to that found in servers and overhead heat exchangers. The ODF and OUF represented 0.15% and 0.03% of the total, respectively. Silva-Llanca et al. [14] estimated an Airside Entropy Generation ${\dot{S}}_{\mathrm{gen},\mathrm{airside}}$ of 34% with respect to the total, demonstrating that the Airside may significantly improve the system efficiency.

We reiterate that the scope of this work ignores potential contributors to the Entropy Generation such as pumps, chillers, and cooling towers. Our claims are limited to phenomena occurring inside the room.

**Figure 7:**
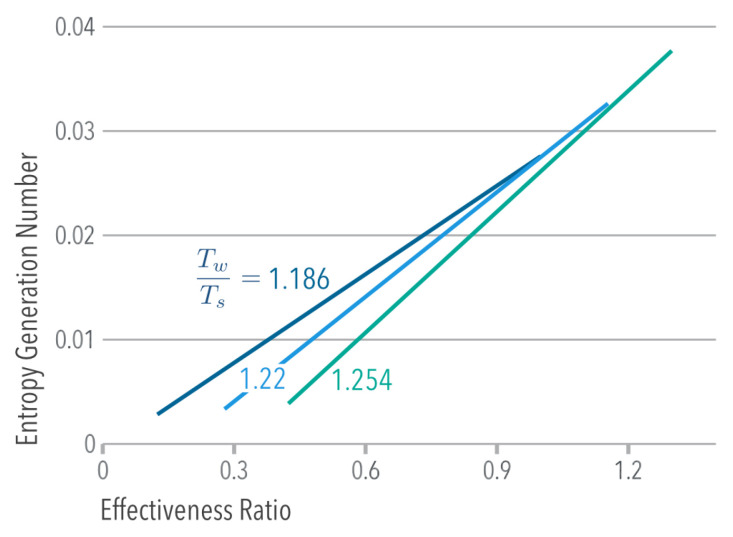
Entropy Generation Number $N_{S}$ as a function of the effectiveness ratio $\xi_{s}/\xi_{\mathrm{HX}}$.

Equation (21) can be re-written in terms of temperatures, heat exchanger effectiveness $\xi_{\mathrm{HX}}$, and server effectiveness $\xi_{s}$, yielding:

(22) $$\begin{array}{ccc} N_{S} & = & \ln\left[\xi_{s}\left(\frac{T_{s}}{T_{s,\mathrm{in}}-1}\right)+1\right]-\ln\left[\frac{T_{w}}{T_{\mathrm{HX},\mathrm{out}}}\frac{1}{1-\xi_{\mathrm{HX}}^{-1}}+\frac{1}{1-\xi_{\mathrm{HX}}}\right]\\ & & +\xi_{s}\left(1-\frac{T_{s,\mathrm{in}}}{T_{s}}\right)\left(\frac{T_{s}}{T_{w}}-1\right) \end{array}$$

where $N_{s}\equiv {\dot{S}}_{\mathrm{gen}}/\left(\dot{m}c_{p}\right)$ is the Entropy Generation Number [24]. Figure 7 indicates that at constant $T_{s}/T_{w}$, the system reduced its Entropy Generation when: (1) the heat transfer in the overhead heat exchangers became more effective (optimized heat exchangers) or (2) when less heat was transferred to the cooling air stream, which contradicts the purpose of a cooling system.

If the containing walls are perfectly insulated and considering the steady state condition (${\dot{Q}}_{s}\;=\;{\dot{Q}}_{\mathrm{HX}}$), Equation (21) reduces to:

(23) $$\begin{array}{ccc} {\dot{S}}_{\mathrm{gen},\mathrm{adiabatic}} & = & \frac{{\dot{Q}}_{\mathrm{HX}}}{T_{w}}-\frac{{\dot{Q}}_{s}}{T_{s}}\\ & = & \frac{{\dot{Q}}_{s}}{T_{w}}\left(1-\frac{T_{w}}{T_{s}}\right) \end{array}$$

or:

(24) $$\begin{array}{ccc} {\dot{X}}_{d,\mathrm{adiabatic}} & = & T_{w}\;{\dot{S}}_{\mathrm{gen},\mathrm{adiabatic}}={\dot{Q}}_{s}\left(1-\frac{T_{w}}{T_{s}}\right) \end{array}$$

where ${\dot{X}}_{d,\mathrm{adiabatic}}$ represents the Exergy Destruction associated with the dissipation of ${\dot{Q}}_{s}$. Equation (24) indicates that the loss of available thermal energy requires reducing $T_{s}$ down to $T_{w}$ in order to avoid increasing the Entropy Generation of the system. Furthermore, the Exergy Destruction would also increase if ${\dot{Q}}_{s}$ is allowed to achieve its maximum value. This value is obtained when both the containing walls are insulated and $\xi_{s}=\xi_{\mathrm{HX}}=1$. Thus,

(25) $$\begin{array}{ccc} {\dot{Q}}_{s,\max} & = & \dot{m}c_{p}\left(T_{s}-T_{w}\right) \end{array}$$

The maximum possible heat transfer in the server allows defining the room cooling efficacy:

(26) $$\begin{array}{ccc} \eta & \equiv & \frac{{\dot{Q}}_{s}}{{\dot{Q}}_{s,\max}}=\frac{T_{s,\mathrm{out}}-T_{s,\mathrm{in}}}{T_{s}-T_{w}} \end{array}$$

According to Equation (26), both the ODF and OUF cooling modes offered similar efficacies (Table 3); the low standard deviations in the results indicate a uniform behavior throughout the servers.

Although small, the advantage (larger η) of the OUF over the ODF mode (Table 3) was consistently correlated with the Entropy Generation technique proposed. Figure 6 depicts a smaller area covered by the Entropy Generation contours in OUF mode; thus, we should expect more efficient Airside convective heat transfer in such a system, as it decreases the overall irreversibilities.

## 5. Unsteady Behavior during the Cooling Process

In this section, we explore the transient response inside the data center room, including servers and heat exchangers. In particular, the dynamic behavior of the Entropy Generation highlights the novelty of this paper for data center literature.

**Figure 8:**
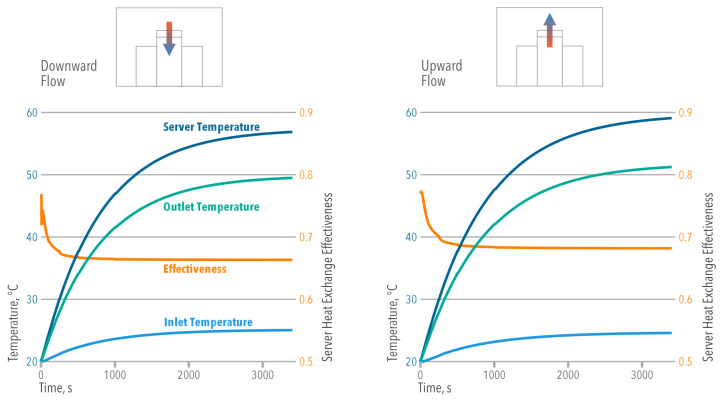
Mean temperature and mean effectiveness evolution in the servers vs. time.

The transient results of Figure 8 indicate that the server effectiveness ($\xi_{s}$) became steady when t>250 s in both cooling modes, reaching the already mentioned values. Even when a higher $T_{s}$ was achieved using the OUF cooling, the greater temperature difference between the server’s exit and inlet increased its effectiveness compared to the ODF cooling. Equation (24) suggests that the lower $T_{s}$ values found in the ODF cooling were responsible for reducing the Entropy Generation or Exergy Destruction during the heat exchange process (Table 2). It is important to understand that the server effectiveness $\xi_{s}$ does not represent a thermodynamic efficiency; therefore, decreasing the Entropy Generation does not necessarily imply a higher η.

**Figure 9:**
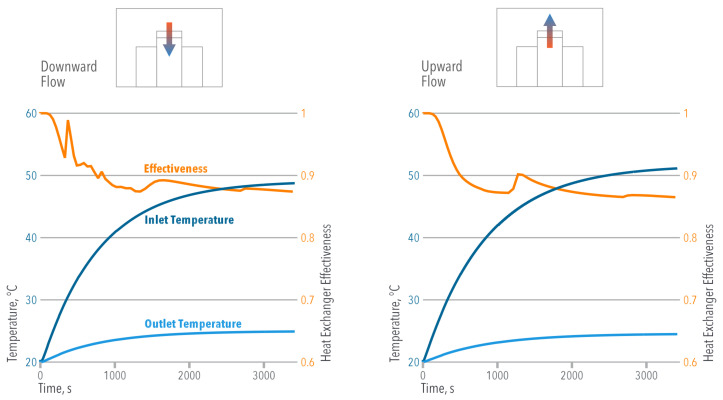
Mean temperature and mean effectiveness in the overhead heat exchangers vs. time.

When evaluating the overhead heat exchangers, Figure 9 shows that they take longer to reach the steady state (t>2750 s). Even though a higher temperature difference ($T_{\mathrm{HX},\mathrm{in}}-T_{\mathrm{HX},\mathrm{out}}$) is found in the OUF cooling, the overhead heat exchanger effectiveness $\xi_{\mathrm{HX}}$ is greater in the ODF cooling.

**Figure 10:**
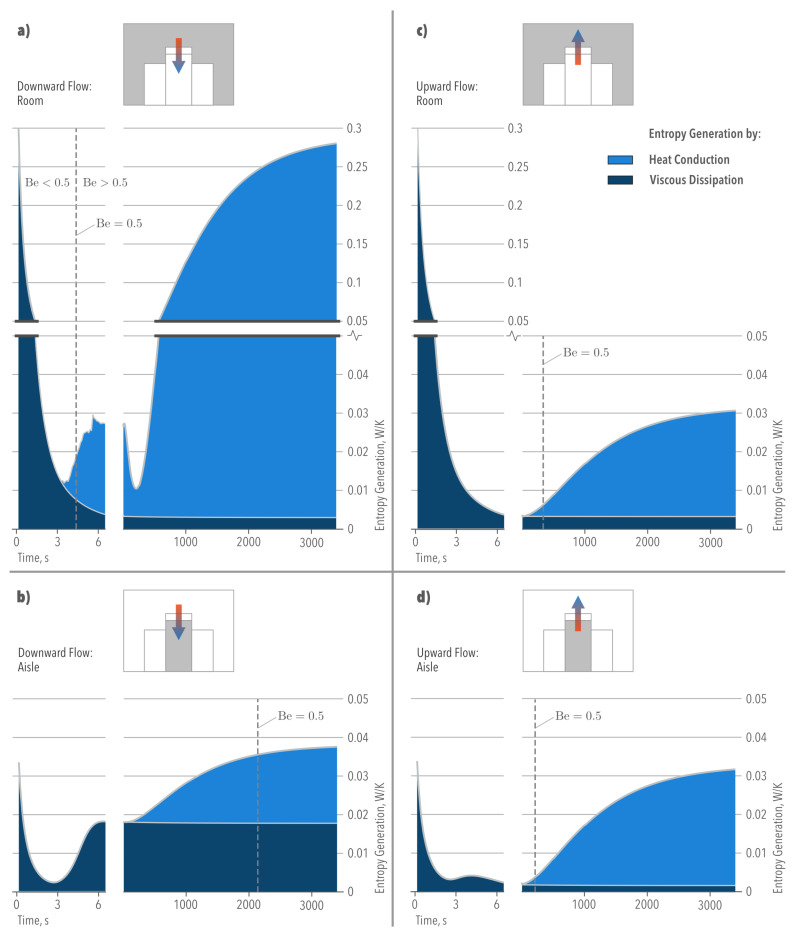
Entropy Generation evolution associated with the Airside (room and aisle).

Figure 10 shows the contribution of fluid mechanics and heat transfer to the increase in the total Entropy Generation of the aisle and room. As observed, and as demonstrated in [14], heat conduction terms were mainly responsible for the increase in ${\dot{S}}_{\mathrm{gen},\mathrm{airside}}$. When ${\dot{S}}_{\mathrm{gen},C}={\dot{S}}_{\mathrm{gen},D}$, both sources of irreversibility (fluid friction and heat transfer) contributed equally to the Entropy Generation. At this point Be=0.5, which is the Bejan number [24] defined as:

(27) $$\begin{array}{ccc} \phi & \equiv & \frac{{\dot{S}}_{\mathrm{gen},D}\left(\mathrm{fluid}\;\mathrm{friction}\right)}{{\dot{S}}_{\mathrm{gen},C}\left(\mathrm{heat}\;\mathrm{transfer}\right)} \end{array}$$

(28) $$\begin{array}{ccc} \mathrm{Be} & \equiv & \frac{{\dot{S}}_{\mathrm{gen},C}}{{\dot{S}}_{\mathrm{gen},C}+{\dot{S}}_{\mathrm{gen},D}}={\left(1+\phi \right)}^{-1} \end{array}$$

Here, if Be<0.5, fluid friction dominates as the main source of irreversibility, whereas when Be>0.5, heat conduction effects dominate.

The relevance of a transient simulation becomes evident in Figure 10, which shows that different irreversibilities dominated along time. Inside the aisle, Figure 10b,d show that both cooling modes offer different advantages in terms of fluid transport and heat dissipation. In the ODF cooling mode, the main irreversibilities were due to viscous dissipation, suggesting higher pumping power requirements. In the OUF cooling mode, irreversibilities were due to the heat transfer process, suggesting a non-uniform temperature distribution. When both cooling modes achieved the steady state, the total Entropy Generation converged to a similar solution. This was not the case for the total Entropy Generation within the room (Figure 10a,c) with higher irreversibilities in the ODF cooling mode (see also Table 2) caused by the heat exchange only.

In practice, data centers are much larger than the physical domain presented here; we ignored key components in the cooling system: pumps, chillers, cooling towers. Still, we offer a novel numerical tool to directly estimate data center thermal management inefficiencies (irreversibilities) in transient scenarios. Dynamic modeling becomes crucial in the ultimate goal of achieving accurate on-demand cooling of data centers.

## 6. Conclusions

In this study, we introduced a numerical model that simultaneously simulates the dynamics of servers, overhead heat exchangers, and the turbulent airflow in an idealized data center room. Based on the discussed results, we conclude the following:

1. The server temperature distributed uniformly throughout the racks. The positioning of the overhead heat exchangers allowed for the cooling airflow to undergo less resistance than the common perforated tile flow. This led to a uniform distribution of cold fluid entering the servers.
2. Aisle containment reduced the Entropy Generation in the Airside to negligible levels when compared to previous results from a legacy data center.
3. Using a second law analysis, we determined the room cooling efficacy, which quantified the deviation of server thermal management from optimum operation.
4. In terms of cooling performance, the OUF and the ODF approaches showed nearly identical results (under the constraints of our idealized analysis); selecting the correct scheme might potentially depend on other aspects, e.g., geometrical, mechanical, economical.
5. The unsteady first and second law metrics presented in this numerical study show promise towards the grand goal of providing instantaneous thermal management as the data center demands it.

## Figures and Tables

**Table 1:** Total entropy generated (W/K) by all the servers and the Airside for each grid size.

	*N* of Nodes	${\dot{S}}_{\mathrm{gen},s}$ (Servers)	${\dot{S}}_{\mathrm{gen},\mathrm{airside}}$ (Room + Aisle)
Mesh 1	108,573	203.5	0.059
Mesh 2	167,493	203.4	0.062
Mesh 3	315,671	203.3	0.073
Mesh 4	563,859	203.9	0.075

**Table 2:** Total Entropy Generation (W/K) for racks, overhead heat exchangers, and the Airside (room-aisle).

	Servers	Overhead Heat Exchangers	Airside
ODF	190.9	17.65	0.318
OUF	203.4	18.55	0.062

**Table 3:** Mean value and standard deviation for the room cooling efficacy.

	ODF	OUF
$\bar{\eta }$	0.66	0.68
$\sigma_{\eta }$	0.0012	0.0006

## References

[1] Hong-Koo N., Song K.S., Chun S.K. The cooling characteristics on the air supply and return flow system in the telecommunication cabinet room. Proceedings of the INTELEC—Twentieth International Telecommunications Energy Conference.

[2] Nakao M., Hayama H., Nishioka M. Which cooling air supply system is better for a high heat density room: Underfloor or overhead?. Proceedings of the Thirteenth International Telecommunications Energy Conference—INTELEC 91.

[3] Sorell V., Escalante S., Yang J. (2005). Comparison of Overhead and Underfloor Air Delivery Systems in a Data Center Environment Using CFD Modeling. ASHRAE Trans..

[4] Cho J., Lim T., Kim B.S. (2009). Measurements and predictions of the air distribution systems in high compute density (Internet) data centers. Energy Build..

[5] Heydari A., Sabounchi P. Refrigeration assisted spot cooling of a high heat density data center. Proceedings of the Ninth Intersociety Conference on Thermal and Thermomechanical Phenomena in Electronic Systems.

[6] Heydari A. Thermodynamics Energy Efficiency Analysis and Thermal Modeling of Data Center Cooling Using Open and Closed-Loop Cooling Systems. Proceedings of the ASME 2007 InterPACK Conference collocated with the ASME/JSME 2007 Thermal Engineering Heat Transfer Summer Conference.

[7] Wu K. (2008). A comparative study of various high density data center cooling technologies. Ph.D. Thesis.

[8] Herrlin M.K., Belady C. Gravity-assisted air mixing in data centers and how it affects the rack cooling effectiveness. Proceedings of the 10th Intersociety Conference on Phenomena in Electronics Systems.

[9] Shah A.J., Carey V.P., Bash C.E., Patel C.D. (2008). Exergy analysis of data center thermal management systems. J. Heat Transf..

[10] Kock F., Herwig H. (2004). Local entropy production in turbulent shear flows: A high-Reynolds number model with wall functions. Int. J. Heat Mass Transf..

[11] Herwig H., Kock F. (2007). Direct and indirect methods of calculating entropy generation rates in turbulent convective heat transfer problems. Heat Mass Transf..

[12] Herwig H. (2012). The role of entropy generation in momentum and heat transfer. Trans. ASME J. Heat Transf..

[13] Zhang L., Lang J., Jiang K., Wang S. (2014). Simulation of Entropy Generation under Stall Conditions in a Centrifugal Fan. Entropy.

[14] Silva-Llanca L., Ortega A., Fouladi K., del Valle M., Sundaralingam V. (2018). Determining wasted energy in the air side of a perimeter-cooled data center via direct computation of the Exergy Destruction. Appl. Energy.

[15] Fouladi K., Wemhoff A.P., Silva-Llanca L., Abbasi K., Ortega A. (2017). Optimization of data center cooling efficiency using reduced order flow modeling within a flow network modeling approach. Appl. Therm. Eng..

[16] Díaz A.J., Cáceres R., Cardemil J.M., Silva-Llanca L. (2017). Energy and exergy assessment in a perimeter cooled data center: The value of second law efficiency. Appl. Therm. Eng..

[17] Bhalerao A., Fouladi K., Silva-Llanca L., Wemhoff A.P. (2016). Rapid prediction of exergy destruction in data centers due to airflow mixing. Numer. Heat Transf. Part A Appl..

[18] Launder B.E., Sharma B.I. (1974). Application of the energy-dissipation model of turbulence to the calculation of flow near a spinning disc. Lett. Heat Mass Transf..

[19] Erden H.S., Khalifa H.E., Schmidt R.R. Transient Thermal Response of Servers Through Air Temperature Measurements. Proceedings of the ASME 2013 International Technical Conference and Exhibition on Packaging and Integration of Electronic and Photonic Microsystems.

[20] Demetriou D.W., Erden H.S., Khalifa H.E., Schmidt R.R. Development of an IT equipment lumped capacitance parameter database for transient data center simulations. Proceedings of the Fourteenth Intersociety Conference on Thermal and Thermomechanical Phenomena in Electronic Systems (ITherm).

[21] Del Valle M., Ortega A. Numerical and compact models to predict the transient behavior of cross-flow heat exchangers in data center applications. Proceedings of the Fourteenth Intersociety Conference on Thermal and Thermomechanical Phenomena in Electronic Systems (ITherm).

[22] Shah R.K., Sekulic D.P. (2003). Fundamentals of Heat Exchanger Design.

[23] Spiga M., Spiga G. (1988). Transient temperature fields in crossflow heat exchangers with finite wall capacitance. J. Heat Transf..

[24] Bejan A. (1996). Entropy Generation Minimization: The Method of Thermodynamic Optimization of Finite-Size Systems and Finite-Time Processes.

